# Targeting deforestation rates in climate change policy: a "Preservation Pathway" approach

**DOI:** 10.1186/1750-0680-3-2

**Published:** 2008-03-03

**Authors:** Kevin R Gurney, Leigh Raymond

**Affiliations:** 1 Department of Earth and Atmospheric Sciences/Agronomy, 550 Stadium Mall Drive, Purdue University, West Lafayette, USA; 2 Department of Political Science, 100 North University Street, Purdue University, West Lafayette, USA

## Abstract

We present a new methodological approach to incorporating deforestation within the international climate change negotiating regime. The approach, called "Preservation Pathway" combines the desire for forest preservation with the need to reduce emissions associated with forest loss by focusing on the relative rate of change of forest cover as the criteria by which countries gain access to trading preserved forest carbon stocks. This approach avoids the technically challenging task of quantifying historical or future deforestation emission baselines. Rather, it places emphasis on improving quantification of contemporary stocks and the relative decline in deforestation rates necessary to preserve those stocks. This approach places emphasis on the complete emissions trajectory necessary to attain an agreed-upon preserved forest and as such, meets both forest conservation and climate goals simultaneously.

## Introduction

With the entry into force of the Kyoto Protocol in February of 2005, the Parties to the United Nations Framework Convention on Climate Change (UNFCCC) have begun to consider how and when developing countries might adopt greenhouse gas emissions reduction commitments [1]. Consideration of developing country participation in the Kyoto framework will need to recognize that the majority of emissions from many developing countries arise from non-industrial emitting activities including deforestation [2,3].

Agreement on the current Kyoto rules were preceded by considerable debate on whether or not deforestation should be included in the emission reduction arithmetic for the first commitment period spanning the years 2008 to 2012 [4-6]. Some argued that nations designating a portion of forest off-limits to deforestation should accrue carbon credits equivalent to the difference between a projection of business as usual deforestation and the protected forest [7,8]. This would achieve much-needed conservation goals while also recognizing the atmospheric benefit of "avoiding" deforestation emissions. Objections were raised to this approach, however, and centered on difficulties such as "leakage" (the displacement of deforestation activity outside the designated preserved forest and stimulated by the preserving activity) and "permanence" (the potentially transitory nature of biospheric carbon due to factors such as pest outbreak or fire) and the likelihood that those credits would be used by developed nations to meet their fossil fuel CO_2 _emissions reductions leading to, at best, a zero sum game from a long-term atmospheric point of view and, at worst, an increase in near-term net greenhouse gas emissions [9-11]. Though deforestation was ultimately not included in the rules for the first commitment period, Parties have indicated a willingness to consider deforestation policy in future negotiating [12]. The negotiations that took place in Bali Indonesia in December of 2007 has reaffirmed this intent and methodological and conceptual work is underway among governments, NGOs, and the scientific community [13].

## Discussion

Thus far, proposals to limit deforestation within the UNFCCC process have followed the model established for limiting fossil fuel/industrial emissions, relying on percentage reduction targets of current deforestation rates relative to an historical or "business-as-usual" baseline [12,14-16]. It is not clear, however, if the baseline/emission reduction model is an appropriate one for the problem of deforestation. To begin with, emissions limits effectively allocate the atmosphere's sink capacity to absorb GHGs – a globally distributed, unowned part of the "global commons" and beyond the control of any individual nation or private actor. Forests and soils, by contrast, are already subject to widely recognized claims of exclusive national control. It seems unlikely, therefore, that a policy designed for an open access resource would be the most appropriate for resources subject to strong existing national claims.

In addition, deforestation is distinctive from fossil fuel/industrial emissions in that the quantity and quality of the unextracted resource (standing forest) is itself associated with social, biological, as well as economic value. For example, the very existence of unextracted coal or oil at particular locations is generally not of direct social or biological concern. In the case of deforestation, however, significant social and biological implications arise when large contiguous forests are reduced to remnant status because forests provide a host of benefits in their unextracted form. These implications extend beyond CO_2 _emissions to include reducing biodiversity, critical habitat, and undiscovered medicinal flora, while potentially compromising the future of local communities dependent upon sustained forest resources.

The analogy between fossil fuel/industrial and deforestation emissions also faces technical difficulties: determining levels of net carbon emissions from forest loss for a base period and a target period is far more difficult than measuring CO_2 _output from fossil fuel consumption and is currently burdened by significant uncertainties [17-19]. Estimates of net carbon exchange in the tropics disagree by factors of two or more and recent work suggests that the differences are likely due to a variety of factors including assumptions about land-use history, land-cover dynamics, and the fate of cleared forest materials [20]. There is an expectation that net carbon exchange estimation in deforesting regions will improve with new remote sensors and renewed international cooperation, but issues of cost, limited historical data, and the challenge of in situ observations remain [21].

In addition, by defaulting to percentage reductions from historical baselines, the analogy fails to recognize the broader set of suggested and actual emissions allocation rules within the global climate change policy process. While percentage reductions from historical baselines were the basis of the Kyoto agreement, many other ideas were proposed and considered during the negotiation process. In actuality, scholars have promulgated a remarkable diversity of rules for distributing emissions entitlements, including schemes based on equal per capita shares, equal shares per unit of energy or economic output, or an auction [22]. Negotiators and policymakers have subsequently considered and utilized many such principles, including in recent allocation contexts like the EU Emissions Trading System [23].

Finally, lowering the deforestation rate may only delay the complete removal of virgin forest rather than preventing it. Only when the deforestation rate approaches zero or forest stands are designated as immune from deforestation pressure, will forest preservation occur. While deforestation rates can of course be adjusted over time in future agreements, that uncertainty leaves forests in some jeopardy. Under this scenario, it is not difficult to imagine a country running out of primary forest before it runs out of incremental deforestation reduction targets.

Allocations of credits starting from a deforestation baseline also risks rewarding countries that have already engaged in substantial harvesting and unfairly punishing countries that have yet to start harvesting their forests at a similar rate. A recent proposal by Danilo Mollicone and colleagues suggests referencing national rates to the global mean deforestation rate, offering a method by which both high and low deforesting countries are offered more equitable incentives [16]. Though a step in the right direction, credit bestowed for preserved forest in one commitment period does not eliminate the possibility that the preserved forest will be removed without penalty in the next. Incremental improvements in the deforestation rate without reference to a fixed goal associated with preserved forest offers only temporary protection. A policy approach that links a rate reduction to a specific quantity of standing stock responds to the unique nature of forests as a contributor to atmospheric CO_2 _and a multi-valued resource in their own right.

These difficulties with the fossil fuel/industrial emissions analogy suggest an opportunity to consider a different approach to deforestation policy. Instead of negotiating deforestation targets relative to historical levels, countries might consider a national target related to the amount of untouched forest they are willing to preserve and the necessary change in deforestation rates required to get there. Such an approach, which attempts to strike a compromise between conservation and emission reduction goals, can be called a "Preservation Pathway".

**Figure 1:**
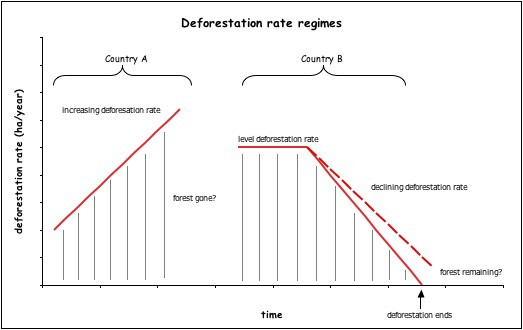
**Preservation Pathways Schematic**. Deforestation rate regime schematic showing examples with two different theoretical countries. Example country A exhibits an increasing deforestation rate with accelerating near-term loss of original forest. Example country B exhibits a constant, then declining deforestation rate with the possibility of preserving a portion of original forest.

For example, many developing countries have experienced increasing rates of deforestation during the last two decades [24]. This represents a positive growth rate in deforestation (equivalent to the second derivative of forest stocks with respect to time or the slope of the deforestation rate) and means that, should nothing change, the date at which the entire original forest removal occurs arrives earlier and earlier as time progresses (Figure 1, example country A). Agreeing to a specific level of preservation would require such a country to transition from an increasing to a declining deforestation rate and follow a trajectory that ensures the preserved amount (Figure 1, example country B).

An important advantage to this approach is that the ability to determine whether a deforestation rate is increasing, level, or decreasing is a relative measure and, as such, can be ascertained reasonably well with a combination of remote-sensing and ground-based measurements. It removes some of the pressure to determine a target level of deforestation that in turn requires absolute estimation of deforestation rates relative to a similarly estimated base year or period. In contrast, the Preservation Pathway approach relies on the ability to estimate relative rates of performance. For example, strategic satellite remote-sensing efforts have achieved good levels of accuracy in assessing the amount of canopy change over time in forested regions [25]. However, translating that into absolute quantities of carbon emitted remains difficult due to the inability of satellites to view near-ground vegetation and below-ground carbon [18]. In situ observations, inverse estimation, and numerical simulation similarly face difficulties in absolute estimation [26,27]. Therefore, determining the percentage change of forest disturbance over a five year period is likely a more robust measure compared to knowing the absolute quantity of carbon emitted over similar periods of time.

The issue of forest degradation – the act of vegetation destruction that is either under the threshold of what is considered deforestation and/or cannot be assessed from remote sensing platforms – remains a challenge for this and all current proposed methodologies. Degradation can raise difficulties if a country has a declining deforestation rate but is simultaneously increasing forest degradation. In order to account for such a circumstance, degradation would have to be estimated with best available methods but like the assessment of forest cover, only in a relative sense, This removes the pressure for absolute historical quantification and places it on the relative trajectory of degradation.

The Preservation Pathway approach is consistent conceptually with how nations treat other terrestrial resources like oil, gas, and coal. It is consistent with a nation's internationally recognized right to consume or protect natural resources located within (or even proximate to) its borders, while encouraging a positive commitment to conserve some of these resources for environmental reasons. In this regard, it reframes developing country contributions with respect to deforestation as a laudable service to the world community, rather than simply a reduction in bad behavior. Such a reframing may be vital to making any such arrangement more politically palatable.

At the same time, it is unreasonable to expect good feelings alone to encourage developing nation's to undertake such commitments. Only when the value of standing forest begins to approach the value of the cleared land for a Soya plantation, one might say, is real progress on this issue likely to occur. Thus, financial incentives are a vital component of any deforestation policy, and this approach will likely require the translation of conserved forests into units of carbon such that value on the international market can be achieved. However, rather than rely on the ability to compute absolute deforestation rates with incremental targets and base year calculations, a post-2012 deforestation trading system could allow countries to sell carbon credits associated with the standing stock of the agreed-upon "preserved forest" once they have transitioned from a positive to negative deforestation growth rate. More specifically, the credits could be based on a combination of the agreed-upon amount of aboveground carbon in the virgin forest to be left intact and the rapidity with which the deforestation rate approaches zero. Though the standing *stock *of carbon in the preserved forest presents some of the same measurement challenges noted for historical baselines, it is different in that it requires an assessment of current conditions only that can be carefully measured and verified (and improved over time). Proposed approaches that require a quantification of baseline or historical deforestation carbon fluxes require a quantification of emission *rates *at past times, a particularly difficult task given the paucity and unreliability of past deforestation rates.

For example, a deforesting country wishing to sell carbon credits in this scheme must establish a target amount of preserved forest within their national boundary. They must further outline the deforestation pathway that ensures the preservation of that forest amount. In order to sell carbon credits at full market value a country would have to meet two objectives: 1) deforestation rates must be declining, and 2) the relative rate of decline from one commitment period to the next (averaged over five year periods) is sufficient to preserve the specified amount of original forest. The quantity of credits awarded would be based on a determination of the amount of carbon in the preserved forest employing standard practices and independent verification (which would also ensure that the preserved forest is not undergoing degradation).

To avoid a flood of credits at the outset of the Preservation Pathway journey, they could be "metered out" over the course of the years prior to achieving the final preservation/zero deforestation point and could scale with the rapidity of deforestation rate reductions or agreed-upon national circumstances. This slow release of credits allows the trading system to maintain integrity and limit price volatility should the estimate of total standing stock of carbon in the preserved forest undergo adjustment (due to improved monitoring, for example), by altering the amount of credits remaining once a country is proceeding down the negotiated path. Should either of the two criteria be violated once begun, the credit value could be discounted or eliminated on an annual basis until the appropriate Preservation Pathway is again achieved. Liability for countries that significantly violate their deforestation rate reduction (such as a reversal from a declining to increasing rate) must be included and could be tied to future trading eligibility.

The future rules could also include emissions targets for developed countries in the post-2012 time period that only allow a fixed fraction of deforestation carbon credits for each reduction performed domestically. This would encourage a market for deforestation credits while continuing to apply pressure for domestic action in the developed world, further diversifying global efforts to reduce greenhouse gas concentrations and making them more robust.

Finally, social, biodiversity and equity criteria could be linked to the crediting system to promote the preservation of continuous versus fragmented forest tracts or particularly valuable forested areas that may contain especially diverse regions or support vulnerable local communities. Research on weighted economic incentives like "agglomeration bonuses" suggests they can be effective policy tools in creating larger and more ecologically sound conservation areas [28]. In a similar manner, a country that chooses a path that leaves a large portion of original forest intact and reduces deforestation rates rapidly with adherence to agreed upon criteria based on equity, social and biodiversity concerns could accrue credits of higher value to sell on rapidly expanding national and international carbon markets.

## Conclusion

The Preservation Pathway approach to including deforestation within international climate change policymaking combines some of the objectives of "avoided deforestation" with objectives that reflect the atmospheric impact of forest removal. It recognizes the value of preserved carbon stocks while incorporating incentives to reduce current deforestation rates and hence, limit the atmospheric burden of carbon dioxide. It creates a more manageable climate policy entry point for many countries in that some of the technical barriers associated with absolute calculations are avoided while emphasizing the long-term goal of changing regimes or pathways of forest loss.

## Competing interests

The authors declare that they have no competing interests.

## Authors' contributions

KRG conceived of and wrote initial draft. LR contributed significant components to final paper. All authors read and approved the final manuscript.
